# South African Flag Sign During Atrial Fibrillation: Physician Recognition of Acute Proximal Left Anterior Descending Artery Occlusion Despite No Automated ST-Segment Elevation Myocardial Infarction Alert

**DOI:** 10.7759/cureus.115282

**Published:** 2026-08-27

**Authors:** Simone Saronni, Mattia Conca, Andrea Agarossi, Alba Ripoll-Gallardo, Riccardo Fiameni, Etrusca Brogi, Riccardo Stucchi

**Affiliations:** 1 Emergency Department, Articolazione Aziendale Territoriale 118, Azienda Socio-Sanitaria Territoriale (ASST), Grande Ospedale Metropolitano Niguarda, Milan, ITA

**Keywords:** automated diagnosis, coronary occlusion, diagonal branch, electrocardiography, left anterior descending artery, myocardial infarction, south african flag sign

## Abstract

The South African flag sign (SAFS) is a non-contiguous electrocardiographic pattern defined by ST-segment elevation (STE) in leads I, aVL, and V2 with reciprocal inferior ST-segment depression (STD), classically associated with first diagonal branch (D1) occlusion. We report a 58-year-old female presenting with acute chest pain and atrial fibrillation (AF), whose 12-lead electrocardiogram (ECG) demonstrated a SAFS phenotype. Automated ECG interpretation generated no ST-segment elevation myocardial infarction (STEMI)-specific alert, classifying the tracing as AF with possible subendocardial injury. This failure may have resulted from AF-induced RR variability combined with non-contiguous STE distribution outside standard algorithmic thresholds. Visual pattern recognition prompted STEMI pathway activation before hospital arrival. Coronary angiography confirmed acute thrombotic total occlusion of the proximal left anterior descending artery (LAD) with Thrombolysis in Myocardial Infarction* *(TIMI) 0 flow. Primary percutaneous coronary intervention restored TIMI 3 flow. The hospital course was complicated by severe left ventricular dysfunction, acute heart failure, and extensive microvascular obstruction. This case confirms SAFS as a rare but clinically meaningful phenotype and provides new diagnostic and angiographic insight by associating it with isolated proximal LAD occlusion, AF, and failed automated STEMI recognition. It suggests SAFS is not restricted to D1 occlusion, and visual ECG interpretation remains essential when automated systems fail.

## Introduction

The South African flag sign (SAFS), defined by ST-segment elevation (STE) in leads I, aVL, and V2 with reciprocal inferior ST-segment depression (STD), maximal in lead III, is a rare electrocardiogram (ECG) pattern mainly associated with acute occlusion of the first diagonal (D1) branch of the left anterior descending artery (LAD) [1,2]. Despite case-report support for this correlation with myocardial infarction (MI), SAFS is not named in current ST-segment elevation myocardial infarction (STEMI) guidelines as an activation pattern.

SAFS should therefore be framed as a visual recognition pattern for acute coronary occlusion rather than a validated culprit-vessel rule [3,4]. Automated ECG interpretation algorithms are generally based on contiguous-lead ST-elevation criteria, although signal-processing and detection approaches differ across platforms [5,6]. As SAFS spans non-contiguous lead groups, it may be prone to under-recognition; whether atrial fibrillation (AF) with irregular RR intervals further hinders recognition is a hypothesis generated by this case.

While SAFS is classically linked to D1 occlusion and has been reported in proximal LAD occlusion within multivessel disease [7], it has never been described in isolated single-vessel proximal LAD occlusion. The present report illustrates SAFS as a rare ECG phenotype and contributes two observations, one angiographic and one diagnostic: the occurrence of SAFS in this setting and the failure of automated STEMI recognition during AF.

## Case presentation

A 58-year-old female with hypertension and gastroesophageal reflux disease (GERD), no previous MI, percutaneous coronary intervention (PCI), cardiac surgery, or arrhythmia, called emergency medical services for severe chest pain. After days of brief self-limited chest pain attributed to GERD, she developed severe persistent pain radiating to both arms.

She was pale, cold, and diaphoretic. Her blood pressure was 110/60 mmHg, heart rate was 156 beats/min with irregular rhythm (Table 1, Panel A), and oxygen saturation was 98% on room air.

**Table 1:** Serial heart rate assessments and ST-segment measurements. Heart rate values were obtained at different time points and by different methods, as expected in atrial fibrillation. ST deviations were measured manually at the J point (TP-segment reference) in complexes with comparable preceding RR intervals, as detailed in the Case Presentation. AF: atrial fibrillation.

Parameter	Assessment	Finding
Panel A - Heart rate		
First contact (08:09)	Pulse palpation	156 beats/min, irregular
Prehospital ECG (08:23)	10-second tracing average	105 beats/min
Automated output (08:23)	Device interpretation	"AF with rapid ventricular response"
Arrival ECG (08:43)	ECG, emergency department	~140 beats/min
Panel B - ST deviation (08:23)		
Lead I	Manual, digital calipers	+1.6 mm
Lead aVL	Manual, digital calipers	+1.8 mm
Lead V2	Manual, digital calipers	+1.4 mm
Lead III	Manual, digital calipers	−3.0 mm
Leads II, aVF	Manual, digital calipers	Reciprocal ST depression

**Figure 1:**
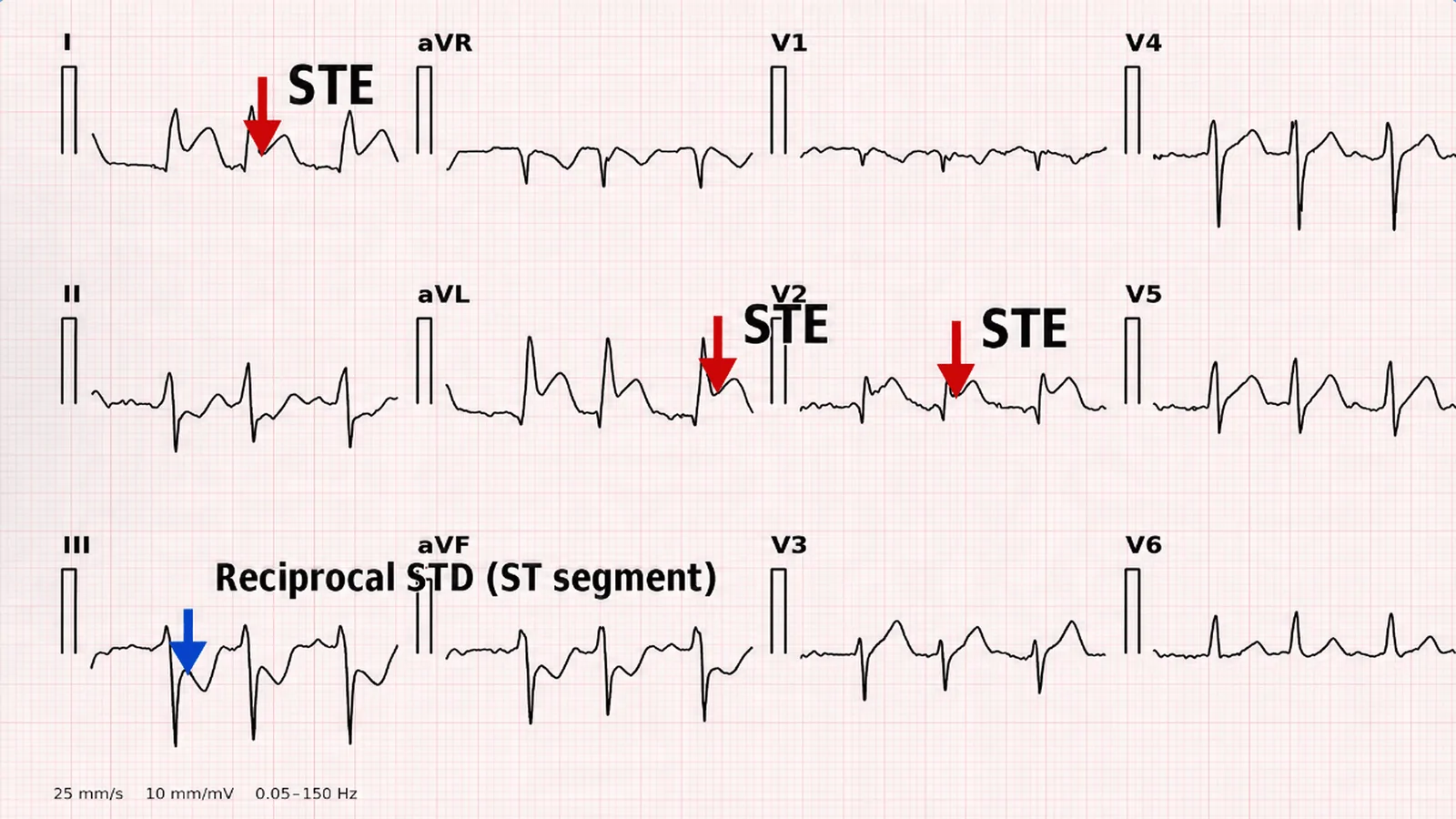
Prehospital 12-lead ECG transmitted telemetrically to the Milan Emergency Dispatch Center (MEDC) demonstrating the South African flag sign. Twelve-lead ECG (25 mm/s, 10 mm/mV) showing atrial fibrillation with irregular RR intervals, ST-segment elevation in leads I, aVL, and V2, and reciprocal inferior ST-segment depression; no automated ST-segment elevation myocardial infarction (STEMI)-specific alert was generated. STE: ST-segment elevation; STD: ST-segment depression.

A 12-lead ECG (Mortara ELI 10, Mortara Instrument, Milwaukee, WI) was recorded at 08:23 and transmitted telemetrically to the Milan Emergency Dispatch Center (MEDC) for remote physician review (Figure 1), which showed AF with a ventricular rate of 105 beats/min, QRS duration of 117 ms, left axis deviation, and a SAFS phenotype (Table 1, Panels A and B).

ST deviations were measured manually by the authors using digital calipers on the telemetrically transmitted tracing at standard calibration (25 mm/s, 10 mm/mV), at the J point with the TP segment as the isoelectric reference; in each lead, representative complexes with comparable preceding RR intervals were selected to limit beat-to-beat variability.

Automated ECG interpretation reported AF with rapid ventricular response, aberrant conduction, left axis deviation, incomplete right bundle branch block, age-indeterminate septal infarction, and ST depression suggesting possible subendocardial injury. The tracing was labeled abnormal and "report not confirmed"; no STEMI-specific alert was generated.

At 08:25, visual review by a trained physician identified the SAFS phenotype and recognized it as a high-risk coronary occlusion pattern. Cardiology teleconsultation was obtained at 08:32, and the STEMI pathway was activated before hospital arrival. She arrived at the hub hospital at 08:43, entered the catheterization laboratory at 08:50, with first wire crossing at 09:15.

On arrival, she remained symptomatic and tachyarrhythmic; a repeat 12-lead ECG in the emergency department documented AF at approximately 140 beats/min with persistent ST-segment elevation in the high-lateral leads and V2 and reciprocal inferior ST-segment depression, confirming ongoing occlusion before catheterization. Echocardiography showed severe left ventricular (LV) dysfunction with ejection fraction (LVEF) of 30-35% and akinesia of the apex, anterior wall, and mid interventricular septum, without signs of acute aortic syndrome or pericardial effusion; a suspected apical thrombus was not confirmed at cardiac MRI. She received antiplatelet and anticoagulant therapy before angiography.

Coronary angiography demonstrated right-dominant circulation and no left main coronary artery disease. The proximal LAD showed acute thrombotic total occlusion with Thrombolysis in Myocardial Infarction (TIMI) 0 flow [8], while the circumflex and right coronary arteries and their branch vessels were unobstructed, consistent with single-vessel coronary artery disease; diffuse mid-to-distal LAD disease was noted. Primary PCI was performed with thrombus aspiration and implantation of a Xience Skypoint 3.0 × 18 mm drug-eluting stent (Abbott, Santa Clara, CA), post-dilated with a 3.25-mm noncompliant balloon. Stenosis was reduced from 100% to 0%, and TIMI 3 flow was restored. The final diagnosis was type 1 STEMI attributable to acute thrombotic proximal LAD occlusion presenting as SAFS [9].

Cardiac MRI on hospital day 3 showed akinesia of the apex, mid septum, and anterior/anterolateral wall, with LVEF of 24%. T2-weighted sequences showed myocardial edema of the mid anterior wall, mid septum, and apex; early post-contrast acquisitions showed extensive microvascular obstruction of the apex; late acquisitions showed transmural late gadolinium enhancement with an ischemic pattern involving the anterior wall, mid septum, and apex. First-pass perfusion showed a fixed anterior deficit. Wall thicknesses were normal, the myocardial salvage index was low, and a mild pericardial effusion was present - findings consistent with a single acute infarction of the entire LAD-dependent territory.

The course was complicated by severe LV dysfunction and acute heart failure requiring dobutamine and levosimendan. Paroxysmal AF was treated with amiodarone. A mild peri-infarction pericarditis with minimal effusion and transient ST-segment accentuation (days 4-8) resolved after a short nonsteroidal anti-inflammatory drug (NSAID) course. Peak high-sensitivity troponin I was 157,273 ng/L, decreasing to 82.1 ng/L at discharge, with N-terminal pro-B-type natriuretic peptide (NT-proBNP) of 4,746 ng/L. Guideline-directed therapy was optimized with sacubitril/valsartan and empagliflozin. Echocardiography showed partial LVEF recovery to 34%. She was discharged after 16 days.

At six months, she was in New York Heart Association (NYHA) class II [10], with no rehospitalizations.

## Discussion

The ECG findings matched published criteria [1,2]. Previous reports linked SAFS to isolated D1 occlusion and high-lateral MI [11,12]. A recent report described SAFS with proximal LAD occlusion, but in multivessel disease, leaving the responsible territory unclear [7]. The present case, with a single isolated proximal LAD lesion, suggests such an occlusion can generate this phenotype.

Cardiac MRI adds a territorial argument. Edema, transmural late gadolinium enhancement, and microvascular obstruction involved the mid interventricular septum and the apex - segments not supplied by the first diagonal branch - indicating that the infarction extended well beyond the diagonal territory. The phenotype itself plausibly reflects dual-territory jeopardy from the single proximal LAD occlusion - diagonal-territory ischemia (ST elevation in I and aVL) coexisting with septal-anterior involvement (V2) - rather than a discrete D1 lesion. Antegrade or retrograde thrombus propagation between the diagonal branch and the LAD cannot be excluded; the documented infarct territory nevertheless indicates acute occlusion of the LAD proper, irrespective of direction. These findings show that, in this patient, SAFS accompanied a large-territory infarction well beyond the classical diagonal distribution; whether this is a general feature of the phenotype cannot be established from a single case. When encountered, the pattern should be interpreted as a signal of possible high-risk acute coronary occlusion rather than a reliable anatomical marker of a specific vessel [3,4]. Proximal LAD occlusion therefore deserves consideration alongside the classical D1 hypothesis, particularly when imaging or clinical features suggest a large territory at risk.

Automated algorithms usually rely on contiguous-lead STE, whereas SAFS ST elevation is non-contiguous. These systems estimate the isoelectric baseline (TP segment) and quantify ST deviation over averaged beats or fixed post-QRS windows; AF-induced RR variability prevents stable baseline detection, so algorithms may misclassify STE as drift, downweight measurements, or default to conservative labels such as "possible subendocardial injury." The mismatch is compounded when STE falls outside contiguous-lead thresholds [5,6], producing a dual (spatial and temporal) blind spot: performance may decline when rare non-contiguous patterns coincide with irregular rhythms. Our case cannot isolate whether AF-related variability, the non-contiguous ST pattern, conduction abnormalities, or platform-specific thresholds were decisive; each remains a candidate mechanism. Automated ECG interpretation should be regarded as a supportive tool; direct physician review remains indispensable, particularly when the clinical presentation is highly suggestive of acute coronary occlusion.

System-level measures may reduce the risk that a clinically evident occlusion is missed when automated interpretation is negative: (1) automated output should be treated as unconfirmed until physician over-read; in our case, mandatory remote over-read within the regional tele-electrocardiogram network (MEDC) is precisely what prevented a missed activation; (2) a "symptom-ECG discordance" escalation rule, whereby a high-suspicion presentation with a negative automated alert triggers immediate expert review rather than reassurance; (3) training in non-contiguous and morphological occlusion patterns, which frequently evade contiguous-lead algorithms; and (4) audit of automated false negatives to identify recurrent blind spots and inform algorithm refinement.

Collaboration between cardiologists and ECG device manufacturers could support prospective validation of pattern-recognition alerts for non-contiguous patterns such as SAFS, complementing contiguous-lead STEMI criteria. Machine-learning approaches trained on curated datasets of angiographically proven occlusions may particularly suit rare patterns and irregular rhythm tracings, in which rule-based algorithms underperform.

This single-case report precludes generalization and warrants strictly hypothesis-generating interpretation. Coronary angiography images are unavailable due to institutional constraints; the complete written reports were used as source data. The report does not specify the occlusion site relative to the first diagonal ostium, and the direction of any thrombus propagation cannot be determined. The arrival ECG and subsequent in-hospital tracings are documented in the clinical records but not publishable as figures. The automated interpretation failure refers to a single tracing on a single platform and cannot be extrapolated to others; prospective multi-platform evaluation is needed.

## Conclusions

In this single-case observation, the SAFS - an electrocardiographic phenotype associated with acute coronary occlusion, not a vessel-specific marker - was associated with an acutely occluded isolated proximal LAD and a large acute anterior myocardial infarction, suggesting, but not proving, that the pattern may occasionally reflect proximal LAD occlusion rather than first diagonal branch occlusion alone. This observation is hypothesis-generating and should be corroborated by further reports before the angiographic differential of SAFS is considered expanded.

In this case, AF and non-contiguous ST elevation coexisted with the absence of a STEMI-specific automated alert; whether AF-related variability contributes to such failures remains a hypothesis requiring prospective evaluation. Automated ECG interpretation should be regarded as a supportive tool; direct physician review remains indispensable, particularly when the clinical presentation is highly suggestive of acute coronary occlusion. A low threshold for direct visual ECG review should therefore be maintained when symptoms suggest myocardial infarction despite no automated STEMI alert - recognition that may support earlier reperfusion activation.
